# Influence of the first wave of COVID-19 on Chinese students’ psychology and behavior: a case study approach

**DOI:** 10.3389/fpsyt.2024.1382301

**Published:** 2024-06-18

**Authors:** Xiudi Zhang, Liang Bian

**Affiliations:** ^1^ School of Education Science, Zhoukou Normal University, Zhoukou, China; ^2^ School of Philosophy and Public Administration, Henan University, Kaifeng, China

**Keywords:** case study, pandemic, psychology and behavior, influence, university students

## Abstract

During the first wave of COVID-19, China demonstrated a strong commitment to epidemic prevention and control. This case study focuses on Z University, which adopted closed management when the epidemic was serious, and examines the influence of COVID-19 on students’ psychology and behavior through interviews with 10 students. The research reveals that while students perceive closed management during the epidemic as enhancing safety and promoting learning engagement to some extent, the epidemic also has adverse effects on their physical health, psychology, and social life. These impacts included deteriorating physical health, feelings of rebellion and depression regarding college life, alongside concerns and aspirations regarding future job stability. In the discussion, we suggest that higher education institutions can utilize this information to shape policies and procedures, particularly concerning mental health and risk communication, not only during the current pandemic but also in future emergency or disaster scenarios.

## Introduction

Universities, for a long time, have played an important role in disseminating knowledge and skills. Universities contribute to the training and development of university students’ knowledge and professional skills to address various scientific and social issues (1). However, previous studies have shown that university students are more likely to have mental health problems (2). University students have just completed high school and entered a relatively unfamiliar environment with its unique set of life and psychological characteristics. For most, they are learning to live independently, meeting new people, and, often, living in close confines with strangers, as well as managing their own finances. Additionally, they may be confronted by challenges posed by a course that may leave them feeling overwhelmed (3). Therefore, to some extent, the prevalence of depressive symptoms among college students is higher than that of the general population or non-college students (4–6).

During the first wave of COVID-19, China was committed to epidemic prevention and control. Based on the severity of the epidemic, China had a series of effective public health measures, such as quarantine of potential cases, monitored tracing of contacted individuals, complete hospitalization of diagnosed patients, and so on (7). In order to protect teachers and students from infection as much as possible, China’s Ministry of Education and Ministry of Industry and Information Technology (8) suggested “suspending classes without suspending learning”. Therefore, students in China made use of different modes of learning, including online learning based on different platforms to achieve this goal. Since the epidemic became severe, universities have also taken many measures. Some universities adopted a closed management approach as an emergency measure to prevent the spread of the virus. Some universities resorted to undertaking innovative online teaching to make sure that students’ learning was going well (9).

Understanding the experience of university students during a global pandemic is crucial, given that the years they spend in university have been indicated as a critical period for life development (10, 11).

According to current research, pandemic experiences can affect student health, study motivation and life satisfaction. Chinese university students attracted significant attention in the fields of public health (12, 13), education (14, 15) and psychology (16–18). A study of 7143 Chinese students has reported that 25% of respondents perceived moderate or severe psychological impact during the COVID-19 pandemic (19). Cao’s (19) research is based on a large population. However, Cao’s study failed to understand the psychological and behavioral changes of students in the specific context of the university implementing closed emergency management during the severe COVID-19 pandemic.

Zhang and Tian (20) found that the challenges Chinese universities faced manifested in the government’s strict management requirements and the students’ demands for freedom of entry and exit. However, Zhang and Tian (20) did not investigate students’ experiences.

In summary, previous research has focused on the manifestation of students’ psychological well-being through anxiety in general and Chinese university challenges, rather than students’ experiences during closed management. Therefore, it is necessary to conduct research on exploring Chinese university students’ coping experiences during closed management.

## Objectives statements

Because of the restrictions, students could not freely go outside of the university. For undergraduate students, they have started the new term in a novel way. This paper aims to investigate the anxiety of Chinese undergraduate students during COVID-19, analyze the impact of the first wave of COVID-19 on undergraduate students, explore whether COVID-19 has increased or decreased their anxiety, and explore the relationship between COVID-19 and student anxiety.

## Materials and methods

### Methodology

To gain a detailed view on the student experience during closed management, a qualitative case study design was used for this study. We adopted naturalistic strategies that paralleled students’ lives and experiences during university closed management, typically interacting with students in a natural and unobtrusive manner (21, 22).

First, case studies are a common way to do qualitative inquiry. The case study not only provides a detailed description of the phenomenon, but also provides an in-depth analysis of the reasons behind the phenomenon, which answers both “how” and “why”, which helps researchers grasp the ins and outs and essence of events. Second, case studies come from practice, without theoretical abstraction and simplification, are a comprehensive and true reflection of objective facts, and taking case studies as a starting point for scientific research can effectively increase the effectiveness of empirical evidence. Third, case studies contain various elements of real-world scenarios, special phenomena, and unexpected phenomena. Indeed, researchers in the case study process may find some causes, phenomena or results and other variables that have not been perceived before, which often become the implicit hypothesis to be tested in the case study, and the basis for future research.

Therefore, this research has drawn attention to the question of what especially could be learned about the case of Chinese students’ stress and personalities during closed management – an instance in time and space.

### Study context

Z University is a provincial-level undergraduate institution with 10 disciplines such as literature, history, economics, management, law, science, engineering, agriculture, education and the arts, as well as 20 colleges (departments) and 65 undergraduate majors. The school has over 20,000 full-time students and 1727 teaching staff. We chose Z University as the research site because it was rated as playing a leading role in pandemic control and prevention at the universities of this city by the municipal government.

From 2020 to the end of 2022, according to the severity of COVID-19, Z University has responded with semi-closed or fully closed management to ensure that students are protected from virus transmission to the greatest extent. Semi-closed management means students can go outside of the campus in the morning and must come back to the campus before 8 p.m. Fully closed management means that students cannot go outside of the campus until the measure is lifted.

### Participants’ rights

We invited participants from a variety of disciplines who were studying at Z University. We send volunteer forms to the school advisors who let the potential participants know about this research. As soon as the person invited had read the Participant Information Sheet and had given their informed consent to participate in this study, we then scheduled a suitable time and method to conduct the interviews. In the data collection, the participants may refuse to answer any questions and are free to leave without giving a reason. All participants have the right to have the audio-recorder turned off at any stage. The participants are entitled to withdraw interview data at any time up to December 2022. The participants were given 50 yuan (around 7 dollars) in gift coupons at the end of the interview. Interview data will be kept anonymous and confidential.

### Participant selection

All the interview data were digitally audio-recorded with the informed consent of the interviewees, and the duration of each interview ranged from 40 to 45 minutes. All the face to face interviews were semi-structured, starting with some pre-designed questions, such as, “How has the COVID-19 effected your life and study?”, “What do you think of the closed management” and “Has/have there been change(s) in your personality?” While the participants verbally shared reflections on events or personal views, we also made additional, spontaneous follow-up inquiries to elicit a more detailed explanation of what the respondent had experienced and how they had experienced it in a bid to collect their stories, thoughts, and feelings in detail (23).

According to the interview sampling literature, the ideal cohort size is between 6 and 12 people (24). Most themes in the study were identified within six interviews, and no new codes emerged after conducting 10 interviews (25). A number of new codes were identified in the first eight individual interviews with over 80% saturation (26). Based on these results, we started the interview process and continued until we recognized data saturation to have been reached. After the analysis process, the interview data were gathered from 10 students. The demographic features of the students are shown in **Table 1**.

**Table 1:** Interviewee demographics (n=10).

Number	Interview	Gender	Seniority	Group	Interview data
1	A	Female	2nd year	Arts	2–9-2022
2	B	Male	4th year	Science	12–9-2022
3	C	Female	3rd year	Literature	21–9-2022
4	D	Male	2nd year	Management	30–9-2022
5	E	Female	1st year	Education	8–10-2022
6	F	Male	4th year	Agriculture	13–10-2022
7	G	Female	1st year	History	18–10-2022
8	H	Male	3rd year	Engineering	23–10-2022
9	I	Female	4th year	Law	28–10-2022
10	J	Female	2nd year	Economics	2–11-2022

### Ethical considerations

As the nature of this study required the students to evaluate their views on the closed management, ethical conditions needed to be considered. Addressing ethical issues was not a process that ended with ethical approval by the Z University Ethics Committee (ZKNHPEC 012378). Specifically, we took the interviews at the dining hall with some snacks and drinking which made the students relax and comfortable.

### Text encoding

In this research, we utilized reflexive thematic analysis, a method recognized for its accessibility and theoretical adaptability in interpreting qualitative data. This approach enables the systematic identification and analysis of patterns or themes within a dataset (27). A central tenet guiding our study was the commitment to faithfully represent students’ opinions and experiences, while also acknowledging and addressing the reflexive influence of our own interpretations as researchers. First, we imported the interview data into the NVivo software, and then the imported material was analyzed and coded to establish free nodes, which could then be rooted. Secondary coding of free nodes was produced according to the research direction and purpose. After that, the visualized content was obtained through the software.

The specific operation process is as follows: import the already organized verbatim manuscripts into NVivo 12 Plus software; and using the grounded theoretical research method, encode the verbatim text of the interviews at different levels. Through a summary of the interview outline, the research group divided all coding variables into two categories: external factors and internal factors. These two classes were used as coding level one nodes, and the subsequent factors were level two. The secondary nodes of external factors included Freedom, School, and Class, whereas the secondary nodes of internal factors included Stable, Negative, Study, and Understanding. We focused on highly repetitive text content during the encoding process. The more frequently the text appeared, the more it reflected the universal significance of the respondent’s psychological behavior.

**Figure 1:**
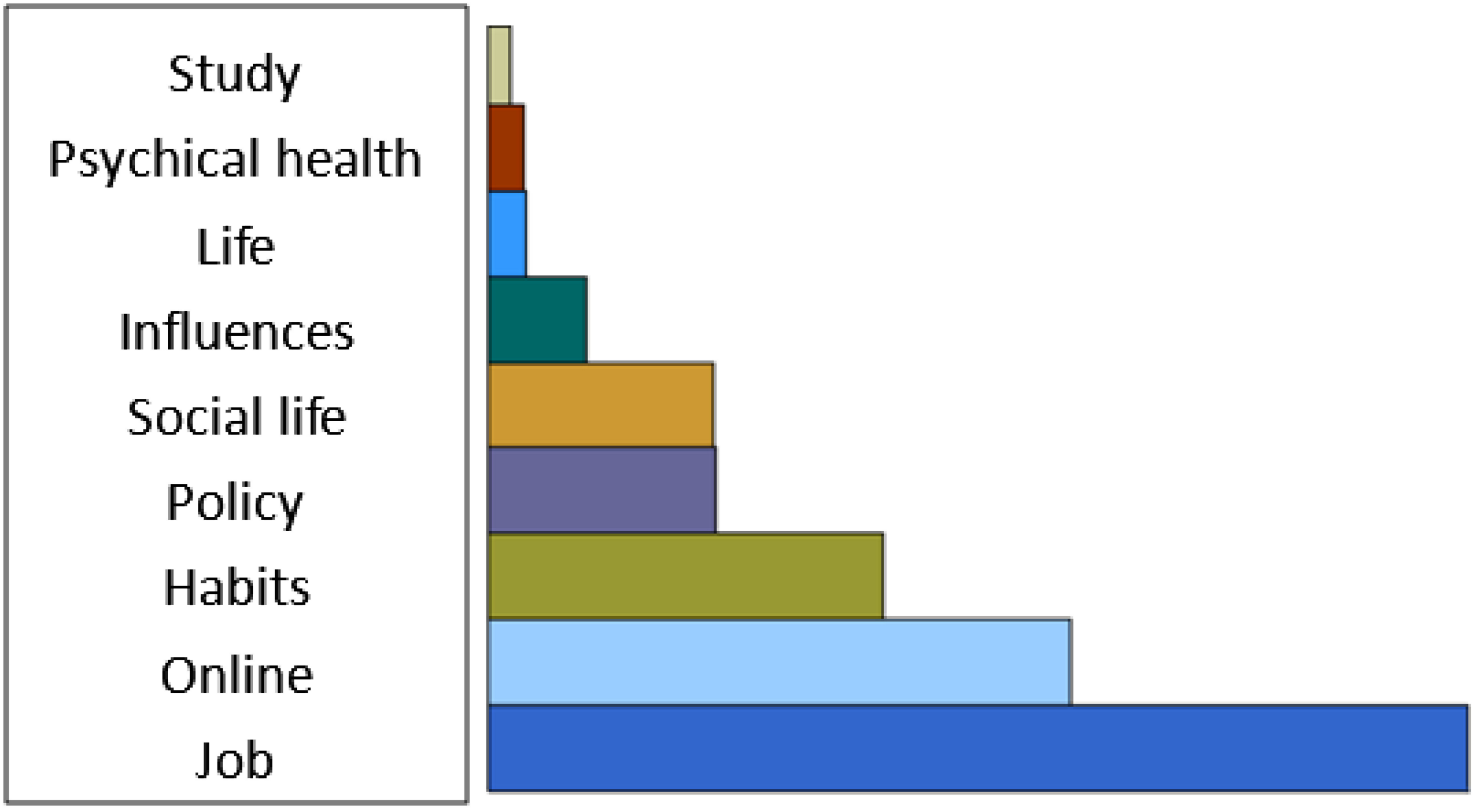
The percentage of the nodes.

Through the scientific encoding of the text, 54 valid coding entries were finally obtained, from which the following nine main nodes could be excavated: online, social life, study, psychical health, life, job, habits, policy and influences. In our qualitative research endeavor, resolving discrepancies among the team regarding nodes was a collaborative and iterative process marked by open dialogue and mutual respect. We approached these discrepancies with a commitment to thoroughness and intellectual honesty, recognizing that diverse perspectives enrich our analysis. Through frequent discussions, we shared our interpretations, allowing for a rich exchange of ideas that often illuminated new insights. After the research group finally sorted the coding results, **Figure 1** was obtained.

From the data in **Figure 1**, it can be seen that during the process of closed management of COVID-19, the students’ responses focused on the following aspects: job intentions, reflections on the closed management, life at the university, online learning quality, and habits, etc.

### The legitimacy of the study

The study’s credibility didn’t hinge on the sheer volume of data amassed but rather on the specific topics distilled through scientific scrutiny of one interviewee’s experiences and insights. Its aim was to gather and scrutinize narratives and perspectives from Z University’s students regarding pandemic closed management. This systematic amalgamation of data and theorization, known as systematic combination (28), allowed for the emergence of cognitive patterns among these individuals, striving for coherence in comprehending all facets of the gathered data (29). Ultimately, the topics were crafted to elucidate life of students within the context of closed management at Z University and to delve into insights for the post-pandemic period.

## Results

### To be safe or free, that is a question

Colleges and universities have an important position in pandemic prevention and control, and the characteristics of dense populations increase the difficulty of pandemic prevention and control. Relevant departments have issued a number of targeted policies, especially the “Technical Plan for the Prevention and Control of the COVID-19 Epidemic in Colleges and Universities (Fourth Edition)” released in August 2021. This plan puts forward detailed requirements for the school management measures of colleges and universities, and emphasizes that under the guidance of local health departments and disease control institutions, emergency measures such as closed management would be taken for universities with epidemic cases or close contacts depending on the situation. Interviewees from the research responded to these matters.

*Student I: I think the university closed management makes me feel safer. Without this, we may also face the same serious risk of the epidemic as other places. The closed management brings the greatest guarantee to our lives – this is a better place. And then, the bad thing about it is that we can’t go out of school freely.*

Students A and B also think they felt safe in the closed university. However, in the process of closed management, university students must not only adapt to the new changes in comprehensive online teaching, and complete various learning tasks arranged by the school, but they also encounter changes in daily life brought about by the closed environment, which is very prone to producing various negative emotions (30).

*Student D: I usually don’t go out even if we are allowed to, but the kind of psychology that I have now is that I may not go out when you open the school door. But now that you don’t let me go out, I may be a little anxious in my heart.*

*Student E: I feel that there is a psychology of wanting to rebel. That is, I may not go out, but you cannot refuse to let me out.*

*Student H: Being unable to freely enter and exit school has given me a certain sense of oppression. I want to release this stress.*

We further asked H whether they went over the wall.

*Student H: Hahaha. Can this be said? I went over the wall once or twice, because I really couldn’t hold back in the last semester. Hahaha.*

An interesting contribution from Student F is that his anxiety was mostly due to filling out health forms.

*Student F: The university is constantly posting about the epidemic situation. My anxiety was caused by filling out the forms to report my health situation to the university. I am afraid of infection and I am worried about my physical health.*

From the above interview excerpts, we can see that “anxiety” and “stress” are common words when describing students’ states of mind, and they perceive that time has been “eaten” by the pandemic. Student E’s psychology of “wanting to rebel” reflects that if an individual’s freedom is reduced or threatened, they will be motivated to reestablish the lost freedom (31). However, reverse psychology can be used ineffectively as a persuasion technique (32) in the Chinese context. Further, the interviewee H indicated “Can this be said?” He means that he needs to be careful about what he has done – go over the wall. Even though he had done so, he would not like the university to know.

### Living in a gilded settings

The university campus is an environment pursuing educational purposes, and it provides “learning” and “opportunities” so that the individual’s inner potential can flourish (33).

In the interviews, we asked the students to generalize their life at Z University during closed management.

*Student J: I cannot go out of the school. Therefore, I can focus on studying to enrich myself. However, staying at the classroom, library and dormitory every day makes me feel a bit bored.*

*Student C: I feel that it is a pity that I can’t go out freely to understand the culture of this city. I am just studying in the school and I don’t know anything else.*

*Student F: There are not too many recreational activities. We spend a lot of time learning. We go into the evening self-studying every day. When there is no class, we either go to the library to study by ourselves, or play sports – playing badminton is a popular sports activity on campus. It is common to make a booking without a venue. Sometimes we also “nest” in the dormitory to chase drama.*

The pandemic resulted in university settings that are unique given the typical permeability of their boundaries and the groups that make up university life, and the activities within the institution that affect social contact between its members (34). The university has formed its own settings so that students’ activities are limited within the university boundary. Students had to adjust to a “study” life as the COVID-19 pandemic affected their social lives and health. Even the researcher found that it is not easy for students to adapt to university life in response to a sudden pandemic such as COVID-19 (35), and they have suggested some solutions for copying challenges for university leaders (20) and students (35). Based on current research, this paper has revealed that students’ perception of college life during closed management is one of enrichment, yet it is also monotonous and one of regret.

### Walking on thin ice

According to the interviews, a phenomenon we cannot ignore is that university students rethink their job intentions in the closed pandemic environment. Many students might be prone to psychological stress because the COVID-19 pandemic has resulted in financial losses and an extreme lack of the social capital normally gained in daily college life and friendships (36).

*Question: Did you have thoughts about your future job?*

*Student F: The epidemic made a lot of people unemployed, so it seems that the budgeted post position is a relatively stable job.*

*Student G: The epidemic has greatly affected many people’s work and income. I feel that I would like to take the civil service examination to get a job with the government.*

*Question: Work within the system, right?*

*Student B: Yes. Everyone wants to be stable now. It looks like everyone is preparing for the public examination. Whether they can be admitted or not, they just give themselves a chance.*

According to an online survey jointly conducted by the Economic Department and the Social Investigation Center of China Youth Daily, “due to the impact of the epidemic, 60% of the graduates surveyed have ‘stabilized’ in employment”, which shows that fresh graduates will also change their employment needs and employment orientation according to changes in the employment situation in the face of the impact of the pandemic. Previous studies have also confirmed that natural disasters or major crises can weaken an individual’s risk appetite and change their attitudes and career choices.

*Question: Do you want to have a stable life?*

*Student I: Yes, when there wasn’t an epidemic in the past, I thought a lot and felt that I could do anything, and I had the idea of whimsically thinking about what kind of work to do, but now, I would not have that kind of thinking.*

*Student A: Of course, yes. Now I think it’s good to be alive. Many shops closed. I felt a little pressure from them. I want to go back to my hometown to work and live.*

According to previous research, McFarland (37) found that the pandemic created instability related to students’ current employment. But in this present study, we found that the pandemic also impacts students’ future employment. More particularly, students are changing their conceptualization of employment from the development of the industry to valuable and guaranteed companies and positions. This also means more people are considering returning to their hometown. On the positive side, the pandemic has accelerated the personal growth of university students and make preparations for personal growth.

### Running on empty

From April to June 2022, Z University implemented the fully closed management, which meant students had online classes in the school and the teachers taught online at home.

*Question: Do you think online teaching has any impact on your health?*

*Student E: For me, it seems like my myopia has increased. Then, I sit for a long time. There may often be some pain in the waist or something.*

*Student B: It’s myopia, and then I gained weight. I don’t move and then I go to bed very late.*

Virtual learning has inevitably increased the amount of time students spend on digital devices every day. Online learning has also affected the physical activity levels of students. Not walking between classes has made some students stationary for hours on end in front of their computers. The public health consequences stemming from a pandemic can be both wide-ranging and long-lasting, affecting not only the most vulnerable, but also leaving a mark on the next generation in profound ways (38). This being said, not every student has issues with health. For example, Student I learned sports skills during the closed management.

*Student I: I was a person who especially disliked sports before. But recently, it has becoming boring. Before university closed management, I certainly was not able to exercise as I would go out to shop on Saturdays and Sundays. Now, I think I cannot go out so I have time to exercise by learning a sport inside the university.*

Generally, with closed management, students’ living environment has become relatively singular, and they are prone to overuse of electronic products and immersing themselves in virtual worlds, which will lead to poor physical health. As the main avenue for learning activities during the COVID-19 pandemic, long hours spent learning with digital devices affect students’ vision, and long hours sitting are associated with a higher likelihood of poor health status among students in the study sample.

At the end, we categorized the above four sub-themes, which can be framed by the three main themes: physical, emotional and intellectual dimensions, as well as social dimensions as shown in **Table 2**.

**Table 2:** Themes of the influence of COVID-19.

Sub-Themes	Main Themes
To be safe or free, that is a question	Physical
Running on empty	
Living in a gilded settings	Emotional and intellectual
Walking on thin ice	Social

## Discussion

Despite the positive achievements of the University in managing the outbreak, such as students feeling safe and aware of the importance of health, it is grateful to the Chinese government. COVID-19 has caused stress related to students’ psychological and physical health. Previous research (35, 39–41) revealed that students leaving campus reported stress, depression, loneliness, lack of motivation, difficulty focusing on schoolwork, restless sleep, appetite changes, job loss concerns, and difficulties coping. To be more specific, this study mainly focused on students who were at the university implementing closed management.

Generally, the university period is an important turning point for the socialization of interpersonal relationships. Social interaction is a vital part of university life, and it is also a critical element in the adaptability of university students to integrate into the new environment in another city (33). This is not only conducive to a deeper exercise of university students’ social abilities, but also conducive to the enrichment of university students’ learning lives.

In a practical way, the university environment itself serves as a crucial platform for students to develop and refine their social skills through diverse interactions with peers, professors, and other members of the campus community. Engaging in group projects, extracurricular activities, and campus events offers opportunities for students to enhance their communication, collaboration, and leadership abilities, which are vital for navigating various social settings beyond academia.

Moreover, the quality of social interactions within the university setting can significantly impact students’ overall well-being and academic success. Building strong social support networks fosters a sense of belonging and connection, reducing the feelings of isolation and stress commonly experienced by students. Access to resources such as counseling services, peer mentoring programs, and inclusive campus initiatives further contributes to the development of students’ social resilience and emotional intelligence. Additionally, cultivating cultural competency and empathy through exposure to diverse perspectives and experiences enriches students’ social awareness and interpersonal skills, preparing them for success in an increasingly globalized and interconnected world. Ultimately, investing in initiatives that promote positive social engagement and inclusivity not only enhances students’ university experience but also equips them with invaluable social competencies essential for their personal and professional growth beyond graduation.

However, due to the closed management, students in this research devoted more time to learning and received more attention. Their offline social networks are obviously smaller than before. The closed management has a certain influence on the physical and mental health of students. Many students inexplicably felt exhausted, and some even explicitly stated that their physical health had been compromised. In the interview surveys, some students stated that during the university closed management period, they sat for a long time and used their phones and computers for extended durations. Therefore, they developed symptoms of lower back pain and low vision. Young university students have not yet fully matured their minds, and their overall psychological quality and psychological adjustment ability are not yet sound enough (42). As such, the university must pay more attention to the psychological issues of students under the conditions of closed management and provide them with the necessary assistance.

The practice of closed management in universities has also had a certain influence on the future planning of students. Some students have a clearer understanding of their career choices and have clearer plans for their lives. As with early interview surveys, some students stated that due to the pandemic, employment for university graduates has become more difficult, and many jobs have been negatively affected, while civil servants and government-sponsored institutions have been less affected. Therefore, they plan to take postgraduate entrance exams or personnel exams in government affiliated institutions in order to become civil servants, work in government funded institutions, or find other good jobs.

Universities can foster stronger partnerships with industries to create more internship and job placement opportunities tailored to students’ fields of study. Offering career counseling services, resume workshops, and networking events can equip students with the skills and resources needed to navigate the job market successfully. Furthermore, integrating practical, hands-on learning experiences into the curriculum can better prepare students for the workforce and enhance their employability. By aligning academic programs with industry demands and providing robust support services, universities can help students feel more confident and prepared to pursue their desired career paths post-graduation, ultimately enhancing their college experience and future prospects.

## The limitations of this study

This study still has some limitations. Firstly, this is a one-off study. We are unable to report changes over time. It is crucial that, based on the actual situation of the pandemic, the time for each university to adopt closed management is not consistent. Secondly, this study only focuses on a small case study of students at Z University. Future research endeavors may benefit from an expansion in sample sizes to better achieve research objectives and enhance the robustness of findings. Increasing the sample size holds significant promise for improving generalizability.

Furthermore, future research could explore innovative recruitment strategies to reach underrepresented or marginalized groups within the population. This may involve leveraging administration, utilizing online platforms, or implementing targeted outreach efforts to ensure the inclusion of diverse perspectives and experiences. Adopting longitudinal designs or collecting data at multiple time points allows researchers to examine track developmental trajectories and explore changes over time, thereby providing richer insights into the phenomena under research.

## Conclusion

This small-scale study explored students’ experiences of closed management at Z University during the COVID-19 pandemic. This study contributes to our understanding of students’ lives during closed management by highlighting three areas: physical, emotional, intellectual and social, as well as social. By hearing students’ voices related to closed management, this can help universities improve their leadership practices and responsiveness. Universities need to strengthen their communications with students based on the tutor responsibility system of full staff, all-round, and whole-process education. Universities should communicate and cooperate with families and society, and work together to do a good job in the psychologically dynamic monitoring of students. Universities also need to strengthen their capabilities in psychological counseling, psychological assistance, and positive psychological education management and maintenance, so as to enhance the psychological resilience of university students and ensure the maintenance of positive mental health.

## Data availability statement

The raw data supporting the conclusions of this article will be made available by the authors, without undue reservation.

## Ethics statement

The studies involving humans were approved by Zhoukou Normal University Ethics Committee. The studies were conducted in accordance with the local legislation and institutional requirements. The participants provided their written informed consent to participate in this study.

## Author contributions

XZ: Writing – original draft, Conceptualization, Data curation, Formal analysis, Methodology, Investigation, Funding acquisition. LB: Writing – review & editing, Conceptualization, Methodology, Investigation, Visualization, Validation, Funding acquisition. 
